# Addressing knowledge and behavior gaps in breast cancer risks: implications for health promotion and intervention strategies

**DOI:** 10.3389/fonc.2024.1456080

**Published:** 2024-11-14

**Authors:** Magdalene Eno Effiong, Israel Sunmola Afolabi, Shalom Nwodo Chinedu

**Affiliations:** ^1^ Department of Biochemistry, College of Science and Technology, Covenant University, Ota, Ogun, Nigeria; ^2^ Covenant Applied Informatics and Communication Africa Centre of Excellence (CApIC-ACE), Ota, Ogun, Nigeria; ^3^ Covenant University Public Health and Wellbeing Research Cluster (CUPHWERC), Covenant University, Ota, Ogun, Nigeria

**Keywords:** breast cancer, risk assessment, education levels, knowledge, females, Nigeria, prevention

## Abstract

**Introduction:**

The growing incidence and high mortality rate of breast cancer (BC) in Nigeria is attributed to increased risk levels, poor prognosis and late detection.

**Methods:**

This study aimed at identifying education-based disparities in BC risk knowledge, lifestyle/ dietary patterns among females in Ogun state, Nigeria. Questionnaires were used to obtain data from 1135 study participants across various levels of education and analyzed using Epi-info software and Graphpad prism.

**Results:**

The lifestyle/dietary pattern assessment revealed that the participants in the secondary level smoked the most (4.50%), accompanied by high red wine (31.00%), fruits and vegetable (73.00%) consumption. Graduates had the highest antibiotics intake (54.50%) and alcohol consumption (12.00%), the undergraduates were the most physically inactive (63.90%) with the highest consumption of carbonated drinks (73.90%), postgraduates consumed red meat/smoked foods the most (70.70%).

**Discussion:**

The knowledge of BC risk positively impacted carbonated drinks, physical inactivity, smoking, antibiotics and alcohol intake. However, it did not affect family history, red meat/smoked foods, fruits and vegetables consumption. Overall, Education has an impact on the knowledge of BC risks which influences the lifestyle/dietary patterns of females in Nigeria.

## 1 Introduction

The human body is made up of cells with similar genetic makeup and DNA sequences. To guarantee the appropriate functioning of the human body, these cells undergo regulated growth, specialization, and death (1). Cancer, a condition defined by abnormal cell proliferation, affects normal cell function, resulting in uncontrolled growth and death resistance (2). Breast cancer (BC) is the most commonly diagnosed cancer, accounting for an estimated 2.3 million new cases each year globally and ranking fifth in terms of cancer mortality (3). The rising prevalence of BC has been related to a variety of causes and risk factors, including inheritance, nutrition, lifestyle, and environmental toxins (4).

Breast cancer remains a major public health concern worldwide, and Nigeria is no exception. Breast cancer is the most common cancer among women worldwide, putting a significant strain on healthcare systems and communities alike (5, 6). While breakthroughs in therapy have increased survival rates, the emphasis has switched to prevention as a more sustainable and successful approach (7, 8). In Nigeria, the rising incidence of breast cancer is exacerbated by late presentation, with around 70% of cases presenting at advanced stages of the disease. This late presentation forces patients to consider complex therapeutic procedures, which are typically prohibitively expensive and limited. Therefore, prioritizing prevention over treatment appears to be a convincing approach to combating the illness.

Breast cancer prevention involves a wide range of strategies aimed at lowering the chance of acquiring breast cancer. These strategies vary from lifestyle changes and early detection through screening to mitigating environmental and genetic risks (9). By addressing the underlying causes of the disease, prevention provides a proactive approach that may avoid the need for costly and invasive therapies in the future. In a resource-constrained setting like Nigeria, where healthcare facilities and financing may be insufficient to fulfill the demands of treating advanced cases, investing in prevention becomes not only practical but also necessary.

Studies by Gwarzo et al. (10), Effiong et al. (11), Ibitoye et al. (12), Ifediora et al. (13), Sadoh et al. (14), Awogbayila et al. (15), Uruntie et al. (16), Udoh et al. (17), Olayide et al. (18), Isara et al. (19), Awodele et al. (20), and Effiong et al. (21), have evaluated the level of BC awareness and prevention practices among Nigerian women of varying ages. Some studies carried out intervention programs to examine the effect of BC education on reducing BC risks and incidence (16). Regardless, wide disparities exist in the level of BC knowledge especially with regards to BC risk factors which translates into the lifestyle and dietary practices of the populace (22). Lifestyle and dietary practices such as physical inactivity, antibiotics intake, smoking, consumption of red meat/smoked foods, carbonated drinks, fruits and vegetables, among others constitutes modifiable risk factors of BC (23) accounting for over 90% of BC cases, while 10% are caused by heredity. This disproportionality in the underlying cause of BC from modifiable risk factors compared to non-modifiable risk factors, highlights the need for a tailored approach that can identify the gaps between knowledge of BC risks and practice so as to inform better intervention strategies and increase effectiveness.

The first step to BC prevention is increased awareness and education on BC risks and associated factors which has the potential to inform lifestyle/dietary pattern changes and reduced BC incidence (24, 25). However, individual heterogeneity exists, in the capacity to comprehend BC related information and translate into practice. Studies have shown that individuals with higher educational qualification possess higher critical thinking abilities and comprehension levels which can enable them process information and translate into action, compared to individuals with lower educational exposure (26). The level of female education in Nigeria has a very wide range, while some persons are fully educated to the graduate level, others have a low level of education stopping at the primary/secondary levels and a few reaching the zenith of educational achievements into holding postgraduate qualifications such as doctorates and professorship (27). All these influences the approach that will be utilized in creating effective awareness, lifestyle/dietary pattern changes while emphasizing the ineffectiveness of the one size fits all approach in BC prevention. This was further supported by a report by Effiong et al. (21), which highlighted differences in BC awareness and channels of communication across levels of education.

The presence of education-based disparity and its significant influence on breast cancer knowledge and lifestyle/dietary patterns informed the need for this study. This study aimed at identifying education-based disparities in breast cancer risk knowledge, lifestyle/dietary patterns among females in Ogun state, Nigeria with a view of developing tailored approaches, bridge the gaps identified, increase effectiveness and reduce the overall BC incidence.

## 2 Methods

### 2.1 Sampling technique

**Figure 1:**
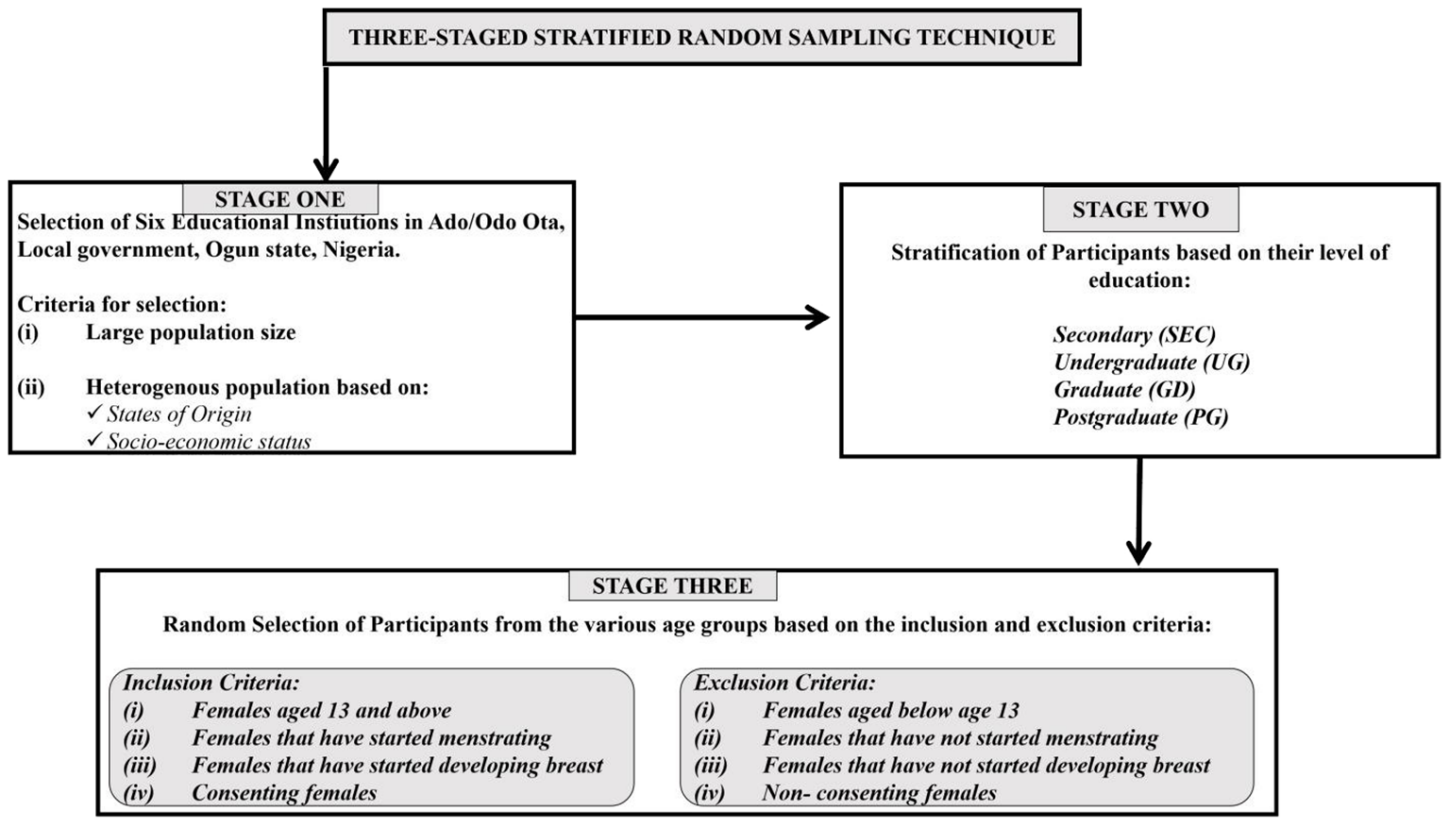
Sampling technique.

This cross-sectional survey was done using a three-stage stratified random sample method. The first stage involves selecting six educational institutions in Ota, Ado-Odo Local Government Area, Ogun State, Nigeria, based on their large and diverse population sizes. The second stage entailed stratifying the participants in the selected institutions based on their levels of education, and the third stage involved picking persons at random from their educational levels based on the inclusion and exclusion criteria shown in **Figure 1**. The inclusion criteria were females aged 13 and up who had started menstruating and had breasts. This study properly followed ethical guidelines, got informed consent from the necessary authorities, and avoided actions that violated confidentiality or constituted a risk to human life. The total population size was 11,350, with a 10% sample size of 1135 people.

### 2.2 Data collection

Questionnaires were utilized as the research instrument, consisting of closed-ended questions with single and multiple correct answers related to the study’s objectives and the Nigerian setting, following the protocols outlined by (13, 28). The questionnaire was divided into three sections: participant socio-demographic information, awareness of breast cancer risk factors, and lifestyle/dietary habits. The questionnaires were distributed physically. The questionnaire’s reliability was tested using the test-retest approach, and its validity was assessed by a health researcher and a demographer.

### 2.3 Statistical analysis

Epi-info, Microsoft Excel, and SPSS version 20 statistical tools were used to collect and evaluate data. Frequencies, percentages, averages, and standard deviations were used to compare the data. The p-values and levels of association were calculated using regression and correlation studies.

### 2.4 Scales of measurement

This study used two measurement scales. There were two assessment scales: knowledge and lifestyle/dietary pattern.

#### 2.4.1 Knowledge assessment scale

A knowledge scale was created to evaluate respondents’ understanding of breast cancer risk. The questionnaire’s knowledge component included four test items with various correct and incorrect answers. The correct answers received one point, while the erroneous options received zero. Overall knowledge scores for each exam question were coded as 100% correct (2 marks), > 50% partially correct (1 mark), and < 50% incorrect (0 marks).

#### 2.4.2 Lifestyle and dietary pattern assessment scale

The respondents’ lifestyle and nutritional habits were evaluated using a frequency-based value assignment scale. The questionnaire’s lifestyle and eating pattern component included nine test items with numerous alternatives for determining the frequency of behaviors. Higher frequencies correlated with higher scores. Options such as never, yearly, monthly, weekly, biweekly, and daily received scores of ‘1’, ‘2’, ‘3’, ‘4, ‘5’, and ‘6’.

## 3 Results

### 3.1 Response rate

The study included 1135 female participants. A total of 1200 questionnaires were distributed; 1135 were completed and returned, yielding a response rate of 94.58%.

### 3.2 Socio-demographic characteristics

**Table 1** depicts the participants’ demographic information, such as age, occupation, religion, marital status and level of education. The participants were from six educational institutions consisting of a university and five secondary schools (two government schools and three private schools). 544 participants (47.93%) were from the university institution and 591 participants (52.07%) were from secondary schools. The participants’ ages ranged from 13 to 60 years, with a mean of 21. Participants were recruited from two types of institutions: 591 (52.07%) were from secondary schools, and 544 (47.93%) were from the university. Participants were broadly divided into two groups: staff and students. 218 (19.21%) participants were staff, consisting of lecturers (4.59%), teachers (82.57%) and non-teaching staff (12.84%). 917 (80.79%) of the participants were students consisting of secondary students (41.88%), undergraduate students (51.25%) and postgraduate students (6.87%).

**Table 1:** Socio-demographic characteristics of study participants across levels of education (n = 1135).

Characteristics	University 544 (47.93)	Secondary School 591 (52.07)	Total 1135 (100)
Age groups			
13 - 19	470 (55.04)	384 (44.96)	854 (75.24)
20 - 30	59 (43.07)	78 (56.93)	137 (12.07)
31 - 40	14 (18.67)	61 (81.33)	75 (6.61)
41 - 50	0 (0.00)	47 (100.00)	47 (4.14)
51 - 60	1 (4.54)	21 (95.45)	22 (1.94)
STAFF AND STUDENTS			
Staff			
Lecturers	10 (100.00)	0 (0.00)	10 (0.88)
Teachers	0 (0.00)	180 (100.00)	180 (15.86)
Non-Teaching Staff (Health workers, Cleaners, etc)	1 (0.18)	27 (4.57)	28 (2.47)
Students			
Secondary students	0 (0.00)	384 (100.00)	384 (33.83)
Undergraduate students	470 (100.00)	0 (0.00)	470 (41.41)
Postgraduate students	63 (100.00)	0 (0.00)	63 (5.55)
LEVEL OF EDUCATION			
Secondary	1 (0.18)	411 (69.54)	412 (36.30)
Undergraduate	470 (86.40)	0 (0.00)	470 (41.41)
Graduate	6 (1.10)	155 (26.23)	161 (14.19)
Postgraduate	67 (12.32)	25 (4.23)	92 (8.11)
MARITAL STATUS			
Single	534 (98.16)	459 (77.66)	993 (87.49)
Married	10 (1.83)	128 (21.66)	138 (12.16)
Divorced/Separated	0 (0.00)	2 (0.34)	2 (0.18)
Widow	0 (0.00)	2 (0.34)	2 (0.18)
RELIGION			
Christianity	544 (100.00)	554 (93.74)	1098 (96.74)
Islam	0 (0.00)	37 (6.26)	37 (3.26)

The participants were drawn from various levels of education, 412 (36.30%) participants had a secondary level qualification, 470 (41.41%) had an undergraduate qualification, 161 (14.19%) had a graduate qualification and 92 (8.11%) had a postgraduate qualification. 12.16% of the participants were married, 87.49% were single, and the rest were widowed and divorced. The participants’ religions were exclusively Christianity (96.74%) and Islam (3.26%).

### 3.3 Knowledge of breast cancer risk factors

**Figure 2:**
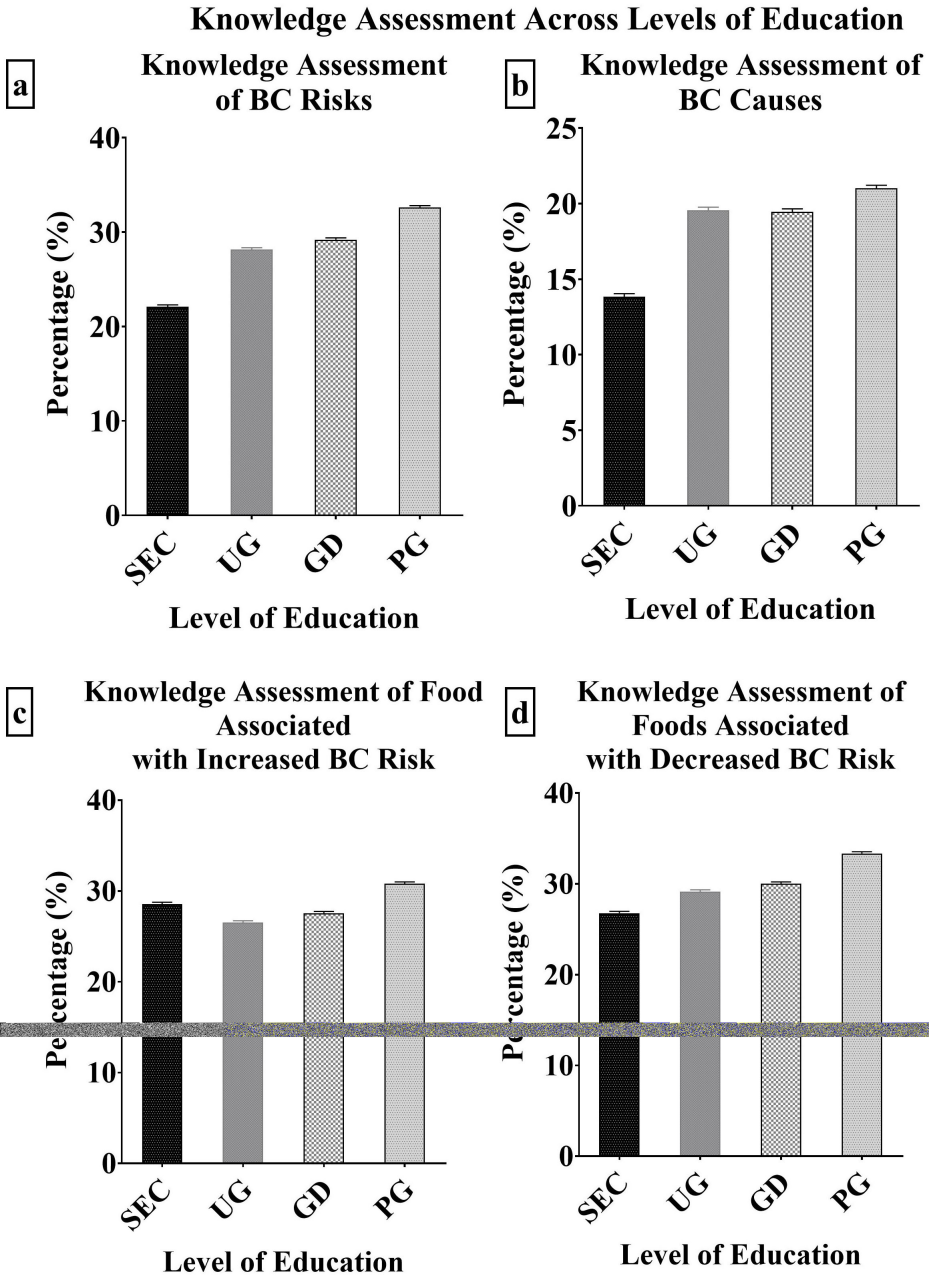
**(A–D)**: Knowledge Assessment of BC risks, causes and foods associated with BC across levels of education.

The knowledge of BC risk factors, causes of BC, foods associated with increased and decreased breast cancer risk was assessed across the levels of educational qualification (**Supplementary Table 2**). The knowledge of various factors consisting of actual breast cancer risk factors and non-risk factors were assessed. The secondary level participants identified radiation (20.87%) and smoking (23.54%) as the major risk factors associated with BC, however, the least identified were early menstruation (0.00%), and late pregnancy (0.49%). The most and least identified BC risk factor was similar among the undergraduates, graduates and postgraduates. Based on the total knowledge score assessment, the knowledge of BC risk factors increased with increase in education. However, there was no significant difference (p < 0.05) in the level of knowledge of BC risk factors across the levels of education (**Figure 2A**).

Knowledge of various causes of breast cancer was assessed across the education levels. The secondary level participants identified always wearing brassiere (44.66%), as the major causes of breast cancer. This was similar to the identified causes by the graduate level participants (32.92%) with heredity (54.66%) as an additional cause. The undergraduates and postgraduates had similar identified major causes of BC. They opted for heredity (48.51% and 60.87% respectively) and poor diet (32.77% and 32.61% respectively). Across all levels of education, the least identified cause of BC was the will of God. The overall, knowledge assessment score revealed that the postgraduates had the highest score (21.02%), while the secondary level participants had the least (13.84%) with no significant difference (p < 0.05) between them (**Figure 2B**).

Across all levels of education, the most identified food associated with increased BC risk was high fatty food and alcohol. The least identified was carbonated drinks by the secondary (12.14%), undergraduates (15.96%) and graduate (21.12%) level participants. The postgraduate participants identified red meat/smoked foods (26.09%) as the least cause of BC. The undergraduates had the least knowledge score of BC foods that increase BC risk (26.52%) while the postgraduates had the highest score (30.80%) with no significant difference (p < 0.05) between them (**Figure 2C**). The knowledge of foods associated with decreased breast cancer risk was also assessed. Fruits and vegetables were the most identified BC risk reducing food by the secondary (55.83%), undergraduates (66.81%), graduates (72.67%) and postgraduates (79.35%). The least identified by the secondary (5.10%), undergraduates (10.85%) and graduates (6.83%) was yogurt, however, the postgraduates opted for milk (8.70%) as the least associated with decreased BC risk. The undergraduates had the least knowledge score of BC causes (26.52%) while the postgraduates had the highest score (30.80%) (**Figure 2D**).

### 3.4 Lifestyle and dietary patterns across age groups

**Figure 3:**
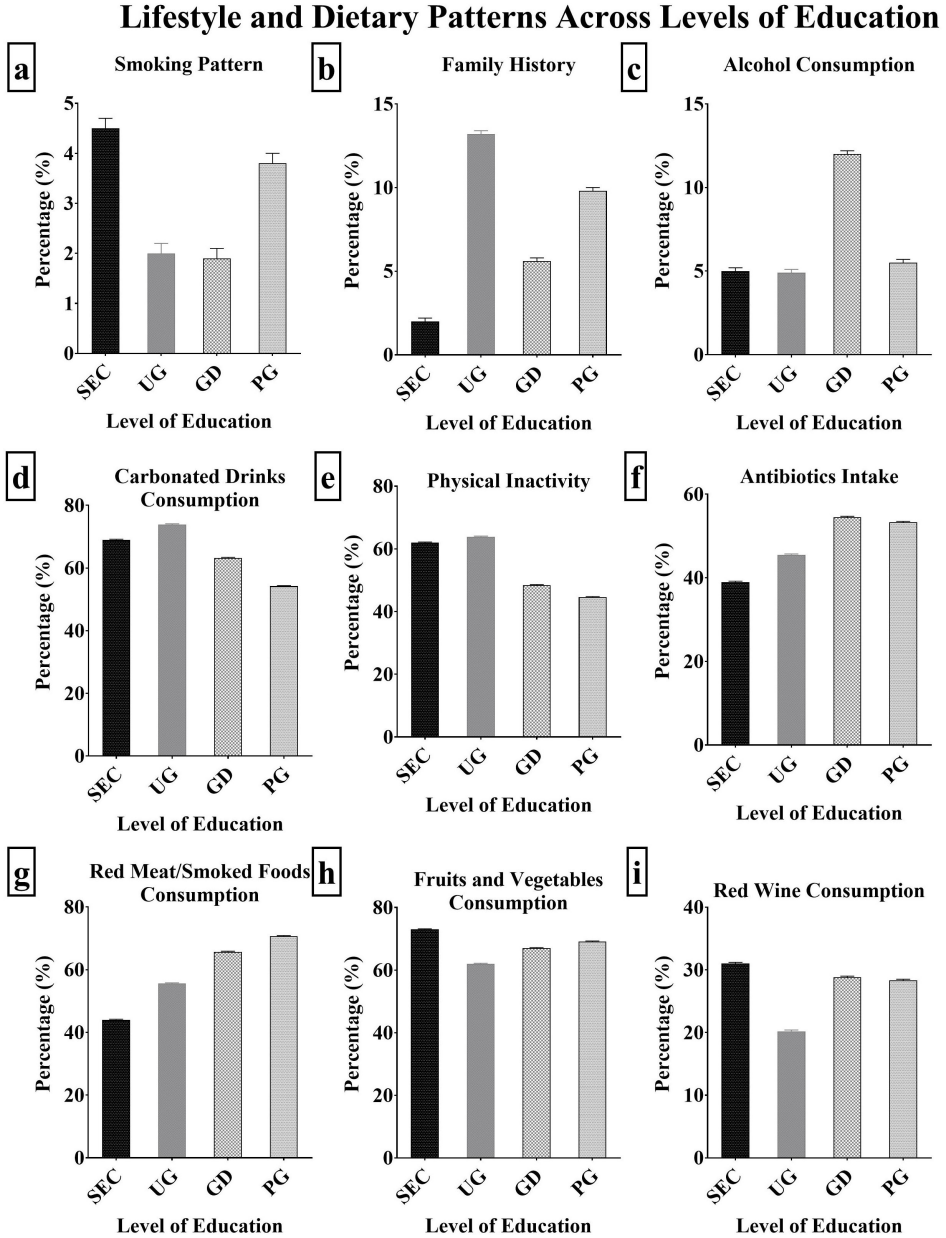
**(A–I)**: Lifestyle/Dietary patterns across levels of education.

The individuals’ lifestyle and nutritional habits that influence breast cancer risk were evaluated (**Figure 3**; **Supplementary Table 3**). The secondary level participants smoked the most (4.50%), followed by the postgraduates (3.80%), undergraduates (2.00%) and graduates (1.90%) (**Figure 3A**). There was no particular trend observed for family history (**Figure 3B**). Graduate level participants consumed the most alcohol (12.00%), followed by the postgraduates (5.50%), while the undergraduates consumed the least (4.90%) (**Figure 3C**). Carbonated drink intake was highest among undergraduates (73.90%), followed by secondary (69.00%), graduates (63.20%) and the lowest among the postgraduates (54.20%) (**Figure 3D**). The undergraduates were the most physically inactive (63.90%), followed by secondary (62.00%), graduates (48.40%) and postgraduates (44.60%) as the least (**Figure 3E**). There was a direct relationship between antibiotic use (**Figure 3F**) and increase in the level of education. Same was observed in the red meat and smoke foods consumption pattern (**Figure 3G**). The secondary level participants consumed the most red wine (31.00%), fruits and vegetables (73.00%) while the undergraduates consumed the least (20.20% and 62.00% respectively) (**Figures 3H, I**).

### 3.5 Impact of BC knowledge on the lifestyle and dietary patterns of the participants

**Figure 4:**
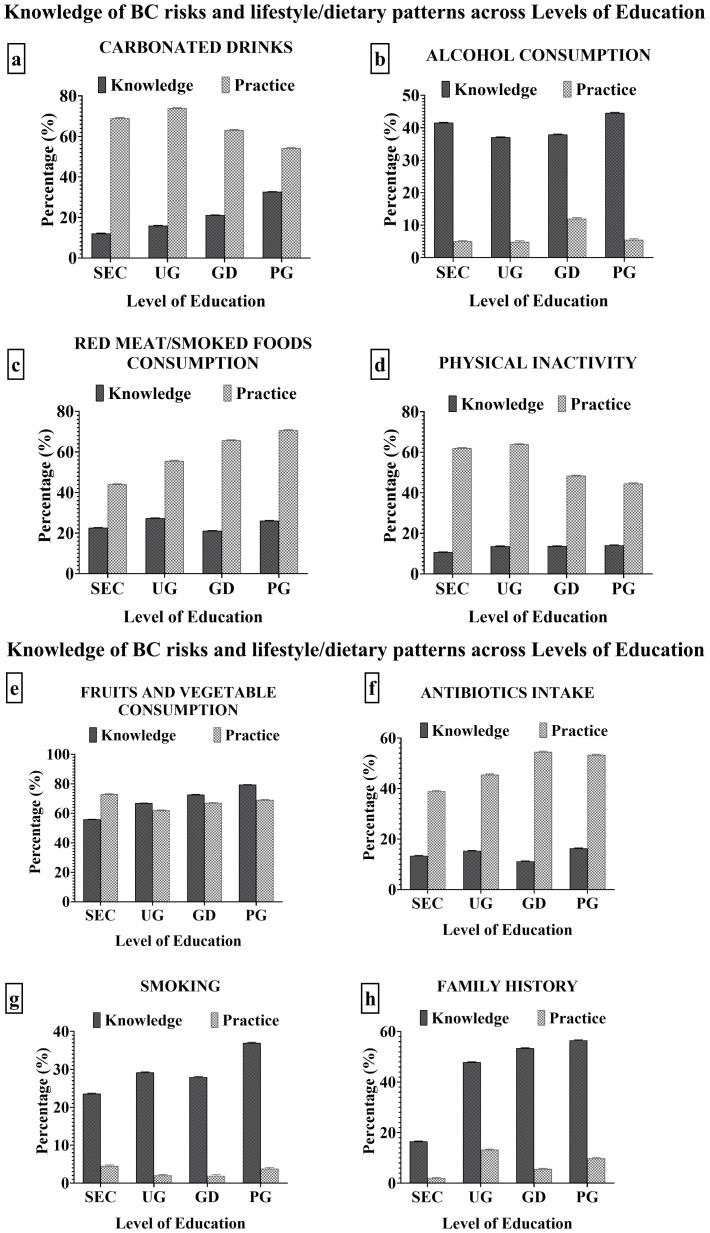
**(A-D)** Impact of BC knowledge on the lifestyle and dietary patterns of the participants. **(E-H)** Impact of BC knowledge on the lifestyle and dietary patterns of the participants.

The effect of BC knowledge on participants’ lifestyle and food patterns was examined across levels of education (**Supplementary Table 3**). The awareness of breast cancer risk factors had a varying impact on the lifestyle and eating patterns of participants (**Figure 4**). The knowledge of BC risk had a direct positive impact on the level of consumption of carbonated drinks (**Figure 4A**), physical inactivity level (**Figure 4D**), smoking (**Figure 4G**), antibiotics (**Figure 4F**) and alcohol intake (**Figure 4B**). The secondary level participants had the least understanding of the effects of smoking (23.54%) and physical inactivity level (10.68%) on BC risk (**Figures 4G, D**), this reflected in their high smoking (4.50%) and physical inactivity (62.00%) compared to other levels of education. The undergraduates had the highest level of carbonated drinks consumption (73.90%) which corresponded to their low knowledge of the risk implication its consumption (15.96%). The graduate level participants had the least knowledge of the implication of antibiotics intake (11.18%) and alcohol consumption (37.89%) on BC risk, which reflected in their high antibiotics intake (54.50%) and alcohol consumption (12.00%). There was no defined impact of the knowledge of BC risk on the consumption of red meat/smoked foods, family history, fruits and vegetables across the levels of education.

## 4 Discussion

Breast cancer is a public health issue that has attracted global attention due to its increasing incidence and fatality rates (29, 30). Breast cancer is related with an aggressive subtype, low survival rates, and a high mortality rate in Nigeria (31). The Nigerian environment has exacerbated the disease’s complexity due to high levels of carcinogen exposure, bad food patterns, and lifestyle (4, 32).

### 4.1 Impact of education on the knowledge of breast cancer risks

Understanding the risk factors for breast cancer is critical for early identification, personalized risk assessment, preventative interventions, and informed decision-making (33, 34). This study evaluated the disparities in the knowledge of BC risk factors and their impact on the lifestyle and dietary patterns across various levels of education. There was a direct positive impact of educational qualification on the knowledge of breast cancer risk factors. Higher educational credentials were found to have a direct favourable impact on knowledge of breast cancer risk factors which was similar to the findings by (21). This could be attributed to more access to resources, higher levels of health literacy, and a better grasp of breast cancer risk factors as educational qualification increases (35, 36). This disparity highlights the need for a targeted intervention with greater focus on lower levels of education.

### 4.2 Lifestyle/dietary patterns across levels of education

Breast cancer risk factors are characteristics that raise a person’s chances of developing the disease (37). Breast cancer risk factors include family history, age, physical inactivity, smoking, a high intake of red meat, processed foods, fizzy beverages, alcohol, antibiotics, and a low intake of fibre, fruits and vegetables, red wine, and so on (21, 38). The lifestyle/dietary patterns of an Individual can be used to adapt recommendations by healthcare practitioners using personalized risk assessment and create preventive actions., such as keeping a healthy weight, exercising regularly, limiting alcohol use and genetic testing, so as to minimize the risk of developing breast cancer (39, 40).

The consumption of red meat and smoked foods are linked to increased breast cancer risks and mortality due to high levels of heme iron, sulphur-containing substances, and mutagens (41, 42), which promote breast carcinogenesis (43, 44), oxidative stress, and reduced gut barrier function (45, 46). The results showed that the consumption of red meat and smoked foods increased with levels of education. This implies that participants at higher education levels are more susceptible to breast cancer via the red meat-induced alterations in the body’s signal transduction and redox homeostasis imbalance. One probable explanation could be socioeconomic factors such as increased income levels associated with higher educational attainment. Persons with higher incomes may have access to a larger variety of foods, including red meat. These findings were not in agreement with Frank et al. (47), and Klink et al. (48), that highlighted that persons with lower educational qualification are more likely to consume red meat than those with higher qualifications.

Alcohol intake raises the risk of breast cancer through hormonal changes, acetaldehyde formation, oxidative stress (49), decreased nutritional absorption, immune system suppression (50), and interactions with other risk factors (51, 52). The results exhibited highest consumption of alcohol among the graduates and least among the participants with secondary school qualification (**Figure 3**). This could be attributable to their enhanced purchasing power, among other things, as evidenced by the employment rate of graduate participants, which was 98.14%, higher than the 51.09%, 0.00%, and 0.00% of postgraduates, undergraduates, and secondary level participants, respectively. Likewise, higher degrees of education may result in higher alcohol use in social and professional situations which exposes people to a variety of social circles that can influence their drinking habits (53).

Smoking is associated with an increased risk of breast cancer, it can change hormone levels, especially oestrogen, disrupting the natural hormonal balance and contributing to the development of breast cancer (54–56). Participants with secondary qualification had the highest level of smoking while the undergraduates and graduates had the least smoking level. This could be as a result of peer pressure, the drive to explore new things, utilization of smoking as a coping mechanisms and lower exposure to anti-smoking efforts and health education initiatives. Individuals may be exposed to anti-smoking efforts and health education initiatives through higher education, which may reduce smoking rates. These findings are consistent with the findings of Adeloye et al. (57),, who showed an overall drop in the level of active smoking in Nigeria, particularly among females.

Physical inactivity has been related to an increased risk of a variety of health problems, including breast cancer (58, 59), through various mechanisms such as hormonal changes, body weight and fat distribution, insulin sensitivity (60, 61), inflammation, immunological function, and biomarker changes (62, 63). Regular exercise lowers circulating oestrogen, a hormone important in the development of breast cancer (64). The American Cancer Society suggests 150 minutes per week of moderate-intensity exercise or 75 minutes per week of vigorous-intensity exercise, as well as muscle-strengthening activities (65). In Nigeria, physical inactivity related deaths have increased by 29% amongst females (66). The results showed that the physical inactivity levels were highest among the undergraduates. This is not agreement with the findings of Adeloye et al. (59), which highlighted that physical inactivity levels are higher among more educated persons. However, the findings of Awotidebe et al. (67), on the decrease in physical inactivity with education levels were consistent with this study.

Antibiotics may raise the risk of breast cancer owing to a variety of causes. These include changes to the immune system, disturbance of the gut flora (68), influence inflammatory reactions and oxidative stress which is critical for overall health (69) The intake of antibiotics across various levels of education was highest among the graduates and least among the secondary level participants. Graduates may have better access to healthcare services, higher health knowledge, and a more proactive approach to obtaining medical care. They may also have more interactions with healthcare experts, increasing their chances of receiving antibiotic prescriptions (70). Higher educational qualifications may also provide more finances to pay for healthcare services and prescriptions (71).

Carbonated drinks, due to their high sugar content, may indirectly increase the risk of breast cancer. Its excessive consumption can lead to insulin resistance and inflammation (72). The results of the study revealed that the undergraduates consumed carbonated drinks the most and the postgraduates the least (**Figure 4B**). There was an inverse relationship between the consumption of carbonated drinks and level of education which is similar to the reports by Okop et al. (73). This was replicated in the results of this study as the undergraduates had the highest carbonated drinks consumption. These dietary choices are influenced by social and peer influences, with undergraduates being more social and peer-oriented, whereas postgraduates may have a different social background or lifestyle (74–76) and stress levels can lead to a desire for comfort foods and beverages (77).

The rapid urbanization in Nigeria grossly affects the level of physical activity, lifestyle and food choices. These makes the consumption of certain healthy and highly beneficial foods to go distinct whilst less healthy, junks and fatty foods takes predominance (66). Red wine, fruits and vegetables are associated with decreased breast cancer risk. Red wine contains resveratrol, a potent anti-carcinogenic phytochemical which reduces breast cancer risk (78). Also, fruits and vegetables contains antioxidants, vitamins, minerals, phytochemicals and fibre (42). These substances boast the immune system and maintains the redox homeostasis, preventing oxidative stress, inflammation and cancer (52). The consumption of red wine, fruits and vegetables was highest among the secondary level participants, compared to other levels of education. In both cases, there were least among the undergraduates. These results are in agreement with the findings of Okop et al. (73). Therefore, a tailored intervention on nutrition education is highly required as reported by Bundala et al. (79). Family history of breast cancer increases a persons chance of having the disease. It involves inheriting a mutation in key breast cancer genes such as BRCA1, BRCA2 etc, which increases breast cancer risk (80, 81), although more education frequently leads to better access to healthcare resources and tests. The family history levels across various levels of education (**Figure 4**) was highest among the postgraduates and least among the secondary level participants. This could be as a result of increased health consciousness and awareness, and those with higher educational qualifications may be more proactive in controlling their health.

## 5 Conclusion

The research findings highlight significant gaps in knowledge regarding breast cancer risks among participants at different educational levels. Specifically, secondary level participants exhibited the least awareness of the effects of smoking and physical inactivity, which corresponded to their lifestyle choices marked by high smoking and low physical activity levels. Undergraduates demonstrated limited understanding of the impact of carbonated drinks consumption on breast cancer risks, reflected in their high intake of such beverages. Similarly, graduate-level participants lacked awareness regarding breast cancer risks associated with antibiotics, alcohol, and red meat/smoked foods, leading to dietary patterns characterized by elevated consumption of these items. Interestingly, postgraduate participants showed the highest knowledge level regarding the risks of red meat/smoked food consumption, yet this knowledge did not translate into healthier dietary habits. Therefore, there is a pressing need for targeted educational interventions aimed at enhancing awareness of breast cancer risks across all educational levels, coupled with strategies to bridge the gap between knowledge and behavior to promote healthier lifestyle choices and reduce breast cancer incidence.

## 6 Recommendation

This study proposes that future research should look into the factors that influence differences in knowledge and behavior about breast cancer risks across educational levels in Nigeria. Qualitative research approaches, such as interviews and focus group discussions, could be utilized to better understand the reasons for various lifestyle and nutritional patterns. A geographically diversified sample, including females from other states or rural areas, would provide a more comprehensive understanding of the impact of cultural, economic, and social variables on BC risk behaviors. Longitudinal studies could look into the long-term effects of educational interventions on lifestyle and nutritional changes.

The findings of this study highlights the importance of customized health promotion efforts suited to different educational levels. These could include raising breast cancer awareness in schools, encouraging frequent screenings and early detection, and integrating community-based programs. Collaboration among government, educational institutions, and healthcare providers is critical for developing long-term interventions that promote healthy lifestyle choices and lower the incidence and mortality rate of breast cancer in Nigeria.

## 7 Ethical consideration

The research team obtained approval from the management of Covenant University to carry out the study. Applications were also made to the participating institutions for their approval to carry out the study. The study was devoid of activities that poses risk or harm to human life and property. The study team utilized the standard research ethics covering informed consent, anonymity of the respondents and their willingness to participate.

## Data Availability

The original contributions presented in the study are included in the article/**Supplementary Material**. Further inquiries can be directed to the corresponding author.
