# Supplementary material for: Addressing knowledge and behavior gaps in breast cancer risks: implications for health promotion and intervention strategies
Source: Front Oncol. 2024 Nov 14;14:1456080. doi: 10.3389/fonc.2024.1456080 (PMC11602397; doi:10.3389/fonc.2024.1456080)
Supplement: Supplementary file 1 [file DataSheet1.docx]

**Sample Questionnaire**

**
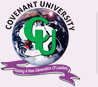
**

**Covenant University, Ota**

**BREAST CANCER RISK KNOWLEDGE, LIFESTYLE AND DIETARY PATTERN SURVEY**

**A project by Covenant University Biochemistry Department**

**QUESTIONNAIRE NO. --------**

**CONSENT AND CONFIDENTIALITY:**

Dear Respondent,

This questionnaire is designed to ascertain the level of knowledge, prevention and examination of breast Cancer. Your answers are strictly confidential and have no legal implications. You are requested to give your honest responses to the following statement and items in the questionnaires. Your responses will be highly appreciated and will be used only for the purpose of this research work.

**Note:** Respondents must be females up to 13 years and have started developing breasts and menstruating.

**Please, kindly tick the appropriate answer to the following questions.**

**Section A: Demographics characteristics**

1. ***Age***: [ ]; Range: 13-19 [ ]; 20-30 [ ]; 31-40 [ ]; 41-50 [ ]; 51-60 [ ];

61-70 [ ]; ≥70 [ ]

2. ***Religion***: (a). Christianity [ ]; (b). Islam [ ]; (c). Traditional [ ]; (d). Others (Specify) ----------

3. ***Marital status***: (a). Single [ ]; (b). Married [ ]; (c). Divorced/separated [ ]; (d). Widow [ ]

4. ***Education (Highest Level)***: (a) None [ ]; (b). Primary [ ]; (c).Secondary [ ]; (d).Tertiary [ ]: (e).Professional [ ] (f) Others (Specify) ...................................;

5. ***Spouse’s education (Highest Level)***: (a) None [ ]; (b). Primary [ ]; (c). Secondary [ ]; (d).Tertiary [ ] (e). professional [ ]. (f) Others (Specify) ...................................;

6. ***Occupation***: (a). Not working [ ]; (b). Self-employed [ ]; (c). Civil/public servant [ ]; (d). Private sector [ ]; Others (Specify) .....................................................…

**Section B: Knowledge of breast cancer risk**

7 ***Which of the following are risk factors for breast cancer? (tick as many that apply)***

(**a**) Early onset of sexual intercourse [ ]
(b) Urinary tract infection [ ]

(c) Family history [ ]

(**d**) Early menstruation (before 11 years old) [ ]

(**e**) Late menstruation (over 16 years old) [ ]

(**f**) Radiation [ ]

(**g**) Increase in Age [ ]

(**h**) Long-term use of birth control pills [ ]

(**i**) physical inactivity [ ]

(**j**) Adequate breast feeding [ ]

(**k**) Smoking [ ]

(**k**) Having many babies [ ]

(**l**) Early pregnancy [ ]

(**m**) Late pregnancy [ ]

8 ***Which of the following are the causes of breast cancer?***

(a) Hereditary [ ]

(b) Always wearing brassiere [ ]

(c) poor diet [ ]

(d) Physical inactivity [ ]

(e) Abortion [ ]

(f) Dense breast [ ]

(g) Will of God [ ]

(h) Attack from the enemy [ ]

9. ***Which of the following foods are associated with increased breast cancer risk?***

(a) Consumption of carbonated drinks [ ]

(b) Consumption of high fatty food [ ]

(c) Consumption of alcohol [ ]

(d) Consumption of red meat [ ]

10. ***Which of the following foods are associated with decreased breast cancer risk?***

(a) Fibre [ ]

(b) Fruits and vegetables [ ]

(c) Vitamins & minerals [ ]

(d) Yoghurt [ ]

(e) Milk [ ]

**Section C: Lifestyle and dietary patterns**

11. ***Which of the following statements relates with your smoking habits and conditions***

(a) I do not smoke [ ]

(b) I smoke [ ]

(c) Someone smokes in my living apartment [ ]

(d) I spend long hours in a smoke filled apartment [ ]

(e) I spend short hours in a smoke filled apartment [ ]

(f) Someone smokes at my workplace [ ]

(g) I spend long hours in a smoke-filled workplace [ ]

(h) I spend short hours in a smoke-filled workplace [ ]

**12. *Which of the following relates to your alcohol habits?***

(a) I do not drink alcohol [ ]

(b) I drink alcohol frequently [ ]

(c) I drink alcohol occasionally [ ]

13. ***Do you drink red wine?***

(a) No, I do not [ ]

(b) Yes, often [ ]

(c) Yes, occasionally [ ]

14. ***Do you take antibiotics?***

(a) No, I never have [ ]

(b) Yes, often [ ]

(c) Yes, occasionally [ ]

(d) Yes, on Doctor’s prescription only [ ]

15. ***Do you consume red meat and smoked foods regularly?***

(a) No, I do not [ ]

(b) Yes, often [ ]

(c) Yes, occasionally [ ]

16. ***How often do you consume fresh fruits and vegetables?***

(a) Never [ ]; (b) daily [ ]; (c) weekly [ ]; (d) biweekly [ ]; (e) monthly [ ]; (f) yearly [ ]

17. ***How often do you consume carbonated (soft) drinks?***

(a) Never [ ]; (b) daily [ ]; (c) weekly [ ]; (d) biweekly [ ]; (e) monthly [ ]; (f) yearly [ ]

18. ***Which of the following describes your physical activity level?***

(a) Sedentary [ ]; (b) Active [ ]; (c) I sleep more than 8 hours a day [ ]; (d) I play sport [ ];

(e) I exercise [ ]; (f) I ride bicycles often [ ] ; (g) I participate in sports [ ], Specify ..............…

**Supplementary Data**

Table 2: **Knowledge of breast cancer risk across levels of education**

|  | **SEC (415)** | **UG (470)** | **GD (161)** | **PG (92)** |
| --- | --- | --- | --- | --- |
| **(a)**  **Knowledge of BC risk factors** |  |  |  |  |
| Early onset of sexual intercourse | 44 (10.68) | 16 (3.40) | 3 (1.86) | 3 (3.26) |
| Urinary Tract Infection | 38 (9.22) | 24 (5.11) | 4 (2.48) | 4 (4.35) |
| Family history | 68 (16.50) | 225 (47.87) | 86 (53.42) | 52 (56.52) |
| Late menstruation (over 16 years old) | 7 (1.70) | 11 (2.34) | 3 (1.86) | 9 (9.78) |
| Early menstruation (before 11 years old) | 0 (0.00) | 0 (0.00) | 1 (0.62) | 0 (0.00) |
| Increase in age | 9 (2.18) | 48 (10.21) | 11 (6.83) | 20 (21.74) |
| Radiation | 86 (20.87) | 277 (58.94) | 71 (44.10) | 53 (57.61) |
| Long-term use of antibiotics | 55 (13.35) | 72 (15.32) | 18 (11.18) | 15 (16.30) |
| Long-term use of birth control pills | 9 (0.00) | 41 (8.72) | 14 (8.70) | 16 (17.39) |
| Physical inactivity | 47 (11.41) | 45 (9.57) | 14 (8.70) | 12 (13.04) |
| Inadequate breast feeding | 30 (7.28) | 5 (1.06) | 5 (3.11) | 2 (2.17) |
| Smoking | 97 (23.54) | 137 (29.15) | 45 (27.95) | 34 (36.96) |
| Having many babies | 5 (1.21) | 9 (1.91) | 0 (0.00) | 2 (2.17) |
| Early pregnancy | 7 (1.70) | 12 (2.55) | 2 (1.24) | 3 (3.26) |
| Late pregnancy | 2 (0.49) | 15 (3.19) | 7 (4.35) | 5 (5.43) |
| **Correct** | 0 (0.00) | 1 (0.21) | 3 (1.86) | 5 (5.43) |
| **Partially correct** | 273 (66.26) | 395 (84.04) | 135 (83.85) | 80 (86.96) |
| **Incorrect** | 139 (33.74) | 74 (15.74) | 23 (14.29) | 7 (7.61) |
| **Mean (%)** | **22.09** | **28.15** | **29.19** | **32.61** |
|  |  |  |  |  |
| **(b) Knowledge of causes of BC** |  |  |  |  |
| Heredity | 95 (23.06) | 228 (48.51) | 88 (54.66) | 56 (60.87) |
| Always wearing brassiere | 184 (44.66) | 156 (33.19) | 53 (32.92) | 27 (29.35) |
| Poor diet | 66 (16.02) | 154 (32.77) | 26 (16.15) | 30 (32.61) |
| Physical inactivity | 44 (10.68) | 64 (13.62) | 22 (13.66) | 13 (14.13) |
| Abortion | 19 (4.61) | 19 (4.04) | 6 (3.73) | 3 (3.26) |
| Dense breast | 65 (15.78) | 88 (18.72) | 15 (9.32) | 7 (7.61) |
| Will of God | 5 (1.21) | 12 (2.55) | 3 (1.86) | 0 (0.00) |
| Attack from the enemy | 71 (17.23) | 104 (22.13) | 16 (9.94) | 7 (7.61) |
| **Correct** | 2 (0.49) | 1 (0.21) | 1 (0.62) | 1 (1.09) |
| **Partially correct** | 167 (40.53) | 274 (58.30) | 92 (57.14) | 56 (60.87) |
| **Incorrect** | 243 (58.98) | 195 (41.49) | 68 (42.24) | 35 (38.04) |
| **Mean (%)** | **13.84** | **19.57** | **19.46** | **21.02** |
|  |  |  |  |  |
| **(c) Foods associated with increased BC risk** |  |  |  |  |
| Carbonated drinks | 50 (12.14) | 75 (15.96) | 34 (21.12) | 30 (32.61) |
| High fatty food | 132 (32.04) | 200 (42.55) | 68 (42.24) | 40 (43.48) |
| Alcohol | 171 (41.50) | 174 (37.02) | 61 (37.89) | 41 (44.57) |
| Red/Processed meat | 93 (22.57) | 128 (27.23) | 34 (21.12) | 24 (26.09) |
| **Correct** | 5 (1.21) | 23 (4.89) | 7 (4.35) | 6 (6.52) |
| **Partially correct** | 343 (83.25) | 328 (69.79) | 119 (73.91) | 73 (79.35) |
| **Incorrect** | 64 (15.53) | 119 (25.32) | 35 (21.74) | 13 (14.13) |
| **Mean (%)** | **28.56** | **26.52** | **27.54** | **30.80** |
|  |  |  |  |  |
| **(d) Foods associated with decreased BC risk** |  |  |  |  |
| Fiber | 72 (17.48) | 138 (29.36) | 43 (26.71) | 27 (29.35) |
| Fruits and vegetables | 230 (55.83) | 314 (66.81) | 117 (72.67) | 73 (79.35) |
| Vitamins and minerals | 94 (22.82) | 264 (56.17) | 63 (39.13) | 49 (53.26) |
| Yoghurt | 21 (5.10) | 51 (10.85) | 11 (6.83) | 12 (13.04) |
| Milk | 41 (9.95) | 75 (15.96) | 12 (7.45) | 8 (8.70) |
| **Correct** | 3 (0.73) | 43 (9.15) | 7 (4.35) | 8 (8.70) |
| **Partially correct** | 325 (78.88) | 325 (69.15) | 131 (81.37) | 76 (82.61) |
| **Incorrect** | 84 (20.38) | 102 (21.70) | 23 (14.29) | 8 (8.70) |
| **Mean (%)** | **26.78** | **29.15** | **30.02** | **33.34** |
|  |  |  |  |  |
| **Overall percentage** | **22.82%** | **25.85%** | **26.55%** | **29.44%** |

**Table 3: Lifestyle and dietary pattern assessment across levels of education**

|  | Questions to assess the Lifestyle/dietary patterns | **Risk score** | **SEC (412)** | **UG (470)** | **GD (161 )** | **PG (92)** |
| --- | --- | --- | --- | --- | --- | --- |
| A | Factors that Increase Breast Cancer risk |  | **F (%)** | **F (%)** | **F (%)** | **F (%)** |
| **1** | **Smoking** |  |  |  |  |  |
|  | Non-smokers | 0 | 378 (91.75) | 457 (97.23) | 155 (96.27) | 86 (93.48) |
|  | Passive smokers | 1 | 31 (7.52) | 8 (1.70) | 6 (3.73) | 5 (5.43) |
|  | Smokers | 2 | 3 (0.73) | 5 (1.06) | 0 (0.00) | 1 (1.09) |
|  | **Mean (%)** |  | **4.50** | **2.00** | **1.90** | **3.80** |
|  |  |  |  |  |  |  |
| **2** | **Family history** |  |  |  |  |  |
|  | No Family history | 0 | 403 (97.82) | 442 (94.04) | 152 (94.41) | 83 (90.22) |
|  | Family history | 1 | 9 (2.18) | 28 (5.96) | 9 (5.59) | 9 (9.73) |
|  | **Mean (%)** |  | **2.00** | **13.20** | **5.60** | **9.80** |
|  |  |  |  |  |  |  |
| **3** | **Alcohol consumption** |  |  |  |  |  |
|  | I do not drink alcohol | 0 | 384 (93.20) | 429 (91.28) | 142 (88.20) | 82 (89.13) |
|  | I drink alcohol occasionally | 1 | 18 (4.37) | 38 (8.09) | 19 (11.80) | 10 (10.87) |
|  | I drink alcohol frequently | 2 | 10 (2.43) | 3 (0.64) | 0 (0.00) | 0 (0.00) |
|  | **Mean (%)** |  | **5.00** | **4.90** | **12.00** | **5.50** |
|  |  |  |  |  |  |  |
| **4** | **Carbonated (soft) drinks consumption** |  |  |  |  |  |
|  | Never | 0 | 27 (6.55) | 30 (6.38) | 6 (3.73) | 10 (10.87) |
|  | Yearly | 1 | 1 (0.24) | 6 (1.28) | 5 (3.11) | 9 (9.78) |
|  | Monthly | 2 | 41 (9.95) | 41 (8.72) | 24 (14.91) | 16 (17.39) |
|  | Weekly | 3 | 165 (40.05) | 161 (34.26) | 72 (44.72) | 34 (36.96) |
|  | Biweekly | 4 | 58 (14.08) | 43 (9.15) | 30 (18.63) | 14 (15.22) |
|  | Daily | 5 | 120 (6.55) | 189 (40.21) | 24 (14.91) | 9 (9.78) |
|  | **Mean (%)** |  | **69.00** | **73.90** | **63.20** | **54.20** |
|  |  |  |  |  |  |  |
| **5** | **Physical inactivity level** |  |  |  |  |  |
|  | Active with exercise | 0 | 92 (22.33) | 60 (12.77) | 34 (21.12) | 34 (36.96) |
|  | Active with no exercise | 1 | 126 (30.59) | 219 (46.60) | 98 (60.87) | 34 (36.96) |
|  | Sedentary | 2 | 194 (47.09) | 191 (40.64) | 29 (18.01) | 24 (26.09) |
|  | **Mean (%)** |  | **62.00** | **63.90** | **48.40** | **44.60** |
|  |  |  |  |  |  |  |
| **6** | **Antibiotics intake** |  |  |  |  |  |
|  | No I never have | 0 | 98 (23.79) | 41 (8.72) | 6 (3.73) | 1 (1.09) |
|  | Yes on doctor's prescription only | 1 | 208 (50.49) | 298 (63.40) | 74 (45.96) | 50 (54.35) |
|  | Yes occasionally | 2 | 38 (9.22) | 67 (14.26) | 57 (35.40) | 26 (28.26) |
|  | Yes often | 3 | 68 (16.50) | 64 (13.62) | 24 (14.91) | 15 (16.30) |
|  | **Mean (%)** |  | **39.00** | **45.50** | **54.50** | **53.30** |
|  |  |  |  |  |  |  |
| **7** | **Red meat and smoked foods consumption** |  |  |  |  |  |
|  | No I do not | 0 | 187 (45.39) | 115 (24.47) | 20 (12.42) | 8 (8.70) |
|  | Yes occasionally | 1 | 88 (21.36) | 201 (42.77) | 73 (45.34) | 38 (41.30) |
|  | Yes often | 2 | 137 (33.25) | 154 (32.77) | 68 (42.24) | 46 (50.00) |
|  | **Mean (%)** |  | **44.00** | **55.60** | **65.70** | **70.70** |
|  |  |  |  |  |  |  |
| **B** | **Factors that reduce breast cancer risk** |  |  |  |  |  |
| **1** | **Fresh fruits and vegetables consumption** |  |  |  |  |  |
|  | Never | 0 | 15 (3.64) | 26 (5.53) | 3 (1.86) | 0 (0.00) |
|  | Yearly | 1 | 0 (0.00) | 11 (2.34) | 0 (0.00) | 0 (0.00) |
|  | Monthly | 2 | 16 (3.88) | 99 (21.06) | 13 (8.07) | 13 (14.13) |
|  | Weekly | 3 | 201 (48.79) | 192 (40.85) | 102 (63.35) | 43 (46.74) |
|  | Biweekly | 4 | 40 (9.71) | 84 (17.87) | 14 (8.70) | 14 (15.22) |
|  | Daily | 5 | 140 (33.98) | 58 (12.34) | 29 (18.01) | 20 (21.74) |
|  | **Mean (%)** |  | **73.00** | **62.00** | **67.00** | **69.10** |
|  |  |  |  |  |  |  |
| **2** | **Red wine consumption** |  |  |  |  |  |
|  | No I do not | 0 | 240 (58.25) | 319 (67/87) | 78 (48.45) | 46 (50.00) |
|  | Yes occasionally | 1 | 94 (22.82) | 118 (25.11) | 75 (46.58) | 37 (40.22) |
|  | Yes often | 2 | 78 (18.93) | 33 (7.02) | 8 (4.97) | 7 (7.61) |
|  | **Mean (%)** |  | **31.00** | **20.20** | **28.80** | **28.30** |
